# ﻿Intruders in beehives? New bee-associated *Ellingsenius* species (Pseudoscorpiones, Cheliferidae) from China based on morphological data and molecular analyses, with comments on pseudoscorpion-bee relationships

**DOI:** 10.3897/zookeys.1234.144259

**Published:** 2025-04-11

**Authors:** Zhizhong Gao, Jianzhou Sun, Feng Zhang

**Affiliations:** 1 Department of Biology, Xinzhou Normal University, Xinzhou, Shanxi 034000, China Xinzhou Normal University Xinzhou China; 2 The Key Laboratory of Zoological Systematics and Application, Institute of Life Science and Green Development, Hebei University, Baoding, Hebei 071002, China Hebei University Baoding China

**Keywords:** China, *COI* gene, phoresy, pseudoscorpions

## Abstract

*Ellingseniusrenae***sp. nov.**, encountered in Guizhou, southern China and the eighth species of the genus, is described and illustrated. An analysis of the *COI* mitochondrial gene (LCO1490/HC02198) confirms the identity of the new species. An identification key to all *Ellingsenius* species is provided, and comments on the pseudoscorpion-bee relationships are included.

## ﻿Introduction

The pseudoscorpion family Cheliferidae Risso, 1827 is nearly cosmopolitan in distribution, occurring on all land masses and many oceanic islands. Although cheliferids are mostly found in leaf litter and under tree bark, some are phoretic on tree-dwelling insects (Harvey 1985) or occur in the nests of vertebrates. Cheliferidaeis divided into two subfamilies, Cheliferinae and Philomaoriinae (Harvey 1992; WPC 2025) and currently includes 312 taxa, including 282 species, nine subspecies, eight nominotypical subspecies, and 12 fossil species, assigned to 64 genera, including five fossil genera (WPC 2025). Only 10 species in six genera have been reported from China (Schawaller 1995; Gao and Zhang 2012; Zang and Zhang 2019; WPC 2025).

The genus *Ellingsenius* Chamberlin, 1932, a member of the subfamily Cheliferinae, was established by Chamberlin (1932) and is widely distributed in the Afrotropical, Indo-Malayan, and Mediterranean regions. There is a single suspect occurrence in the Nearctic. The genus differs from all other genera of the family Cheliferidae in having three galeal setae on the chelicerae (Murthy and Ananthakrishnan 1977). *Ellingsenius* currently includes only seven valid species (Judson 1990; WPC 2025), which are all associated with beehives (Gonzalez et al. 2008).

Pseudoscorpions have been recently reported in colonies of the eastern honey bee (*Apisceranacerana* Fabricius, 1793) in China (Lin et al. 2020); they were assigned to *Ellingsenius* based on a bioinformatics analysis where the sequences were compared to the NCBI GenBank database using the BLAST tool with the MegaBlast algorithm. Although Lin et al. (2020) did not present any morphological data, their phylogenetic study showed that the pseudoscorpions might represent a new species of *Ellingsenius*.

We recently received some pseudoscorpion specimens collected from beehives from Guizhou province, China, which were easily attributed to the genus *Ellingsenius* using morphological criteria. However, we found characters that differed from all known species, and these specimens are here described as *E.renae* sp. nov. Molecular analyses were also performed to clarify the status of the new species. This allows us to describe the *Ellingsenius* species morphologically from China for the first time, which expands the distributional range of the genus.

## ﻿Materials and methods

### ﻿Morphology

The specimens examined for this study are preserved in 75% alcohol and deposited in the Museum of Hebei University (**MHBU**) (Baoding, China). Photographs, drawings and measurements were taken using a Leica M205A stereomicroscope equipped with a Leica DFC550 Camera. Detailed examination was carried out with an Olympus BX53 general optical microscope. Temporary slide mounts were prepared in compliance with the method outlined by Gao et al. (2017). Images were edited and formatted using Adobe Photoshop 2022.

Terminology and measurements follow Chamberlin (1931) with some minor modifications to the terminology of trichobothria (Harvey 1992; Judson 2007) and chelicera (Judson 2007). The chela and chelal hand are measured in lateral view and others taken in dorsal view. All measurements are given in mm unless noted otherwise. Proportions and measurements of pedipalps and carapace correspond to length/width, those of legs to length/depth.

The following abbreviations are used for the trichobothria: *b* = basal; *sb* = subbasal; *st* = subterminal; *t* = terminal; *ib* = interior basal; *isb* = interior subbasal; *ist* = interior subterminal; *it* = interior terminal; *eb* = exterior basal; *esb* = exterior subbasal; *est* = exterior subterminal; *et* = exterior terminal. Cheliceral setae: *gs* = galeal; *es* = exterior; *is* = interior; *sb* = subbasal; *b* = basal.

### ﻿Molecular methods

We extracted total genomic DNA from pseudoscorpion chela and legs using the QIAGEN DNeasy Blood & Tissue Kit (Qiagen Inc., Valencia, CA), following the manufacturer’s protocols with the elution buffer volume used is 60 μl. We used the primer pair LCO1490/HCO2198 (Folmer et al. 1994) to amplify *COI* sequences under the following PCR reaction protocol: initial denaturation at 94 °C for 5 min; 35 cycles of denaturation at 94 °C for 30 s, annealing at 45 °C for 40 s, and elongation at 72 °C for 1 min; and final extension at 72 °C for 7 min. The 25 μl PCR reactions included 12.5 μl of 2×Tag Master Mix (KangWei Biotech, Beijing, China), 0.8 μl of each forward and reverse 10 μM primer, 4 μl of genomic DNA, and 6.9 μl of double-distilled H_2_O. The PCR products were visualized by agarose gel electrophoresis (1% agarose). All PCR products were purified and sequenced at Sangon Biotech (Shanghai, China) Co., Ltd.

Sequence alignments were carried out using MAFFT v. 7.313 (Katoh and Standley 2013) with the L-INS-I strategy, and checked for the presence of stop codons of *COI* by translating them into amino acid sequence using Geneious Prime (Kearse et al. 2012). Ambiguously aligned positions were culled using trimAl v. 1.2 (Capella-Gutiérrez et al. 2009) with default parameters. The final alignment as Suppl. materials 1, 2. The pairwise genetic distances (Kimura 2-parameter K2P) were calculated using MEGA v. 11 (Tamura et al. 2021) to assess the genetic differences (with pairwise deletion option).

Phylogenetic analyses were performed under the assumptions of maximum likelihood (ML) with GTR+I model and Bayesian inference (BI) with GTR model, respectively. The best-fit nucleotide substitution model was tested using ModelFinder (Kalyaanamoorthy et al. 2017) in PhyloSuite v. 1.2.3 software (Zhang et al. 2020). The ML analysis was conducted using IQ-TREE v. 1.6.8 (Nguyen et al. 2015) in PhyloSuite. The robustness was evaluated by 5000 bootstrap pseudo replicates. BI analysis was performed using MrBayes v. 3.2.6 (Ronquist et al. 2012) (2 × 10^6^ generations) in Phylosuite, in which the initial 25% of the sampled data was discarded as burn-in. The remaining trees were used to assess posterior probabilities for nodal support. The resulting trees were visualized and edited using FigTree v. 1.4.4 (Rambaut 2018).

## ﻿Results

### ﻿Taxonomy

**Family Cheliferidae Risso, 1827**

**Subfamily Cheliferinae Risso, 1827**

#### ﻿ Ellingsenius

Taxon classificationAnimaliaPseudoscorpionesCheliferidae

Chamberlin, 1932

F47570A0-DE09-531F-A32F-99AAF13FB6E8

##### Type species.

*Chelifersculpturatus* Lewis, 1903, by original designation.

#### ﻿ Ellingsenius renae

Taxon classificationAnimaliaPseudoscorpionesCheliferidae

Gao & Zhang sp. nov.

81F39EBB-A671-54D9-B83D-A4CBDC8F22CB

https://zoobank.org/3FEDC5B9-6643-46ED-BFA8-6CBD91171D6C

**Figure 1.**
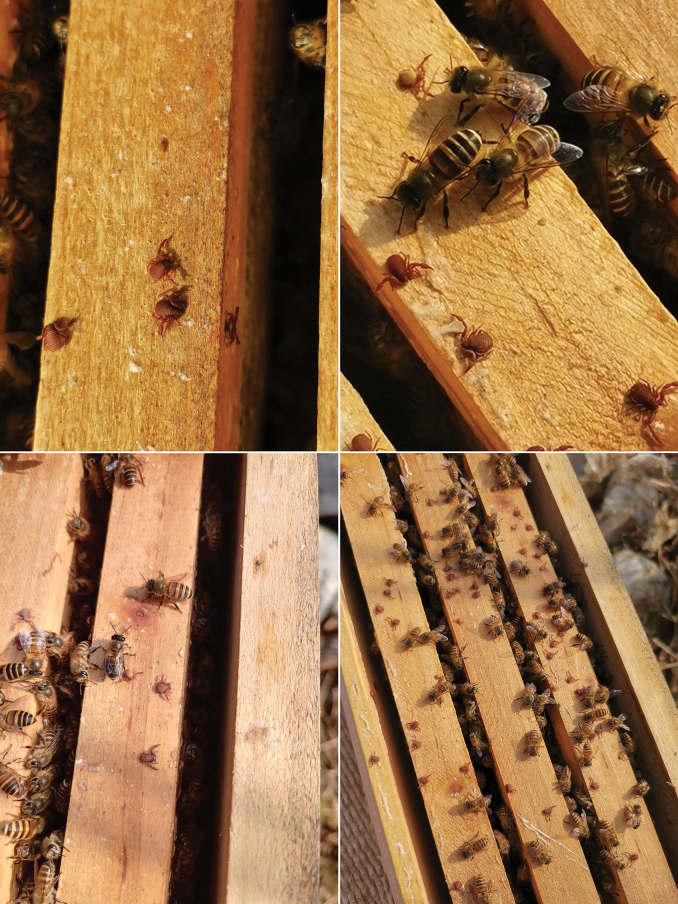
*Ellingseniusrenae* sp. nov., a bee-associate pseudoscorpion, found in bee hives of *Apisceranacerana* in southern China. Photographs by Dr Xiaoxiao Ren.

**Figure 2.**
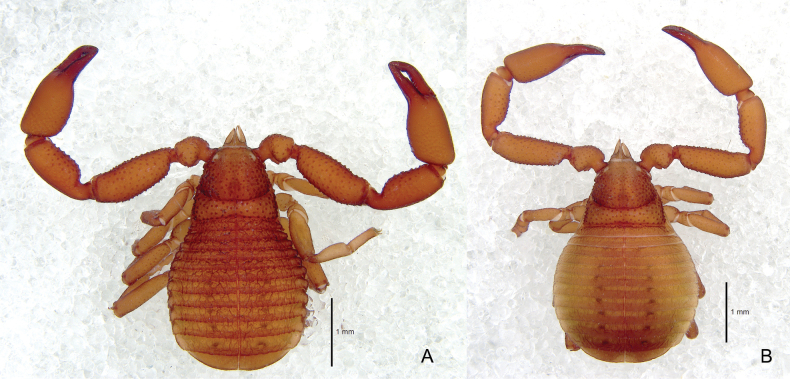
*Ellingseniusrenae* sp. nov. **A** holotype male, dorsal view **B** paratype female, dorsal view.

**Figure 3.**
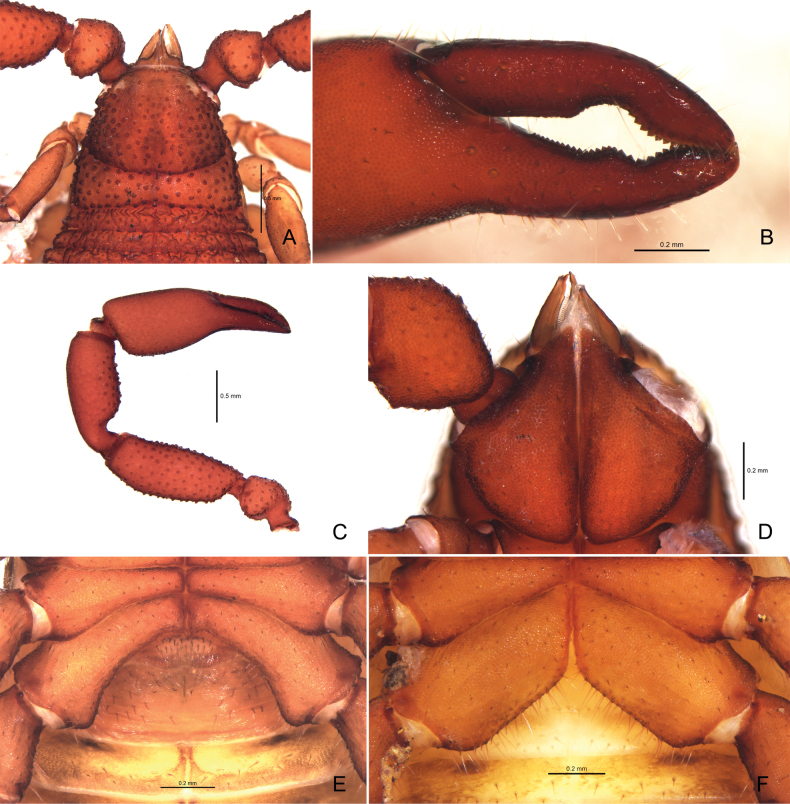
*Ellingseniusrenae* sp. nov. **A–E** holotype male **A** carapace, dorsal view **B** left chelal fingers, lateral view **C** left pedipalp, dorsal view **D** pedipalpal coxa, ventral view **E** genital region, ventral view **F** paratype female, genital region, ventral view.

**Figure 4.**
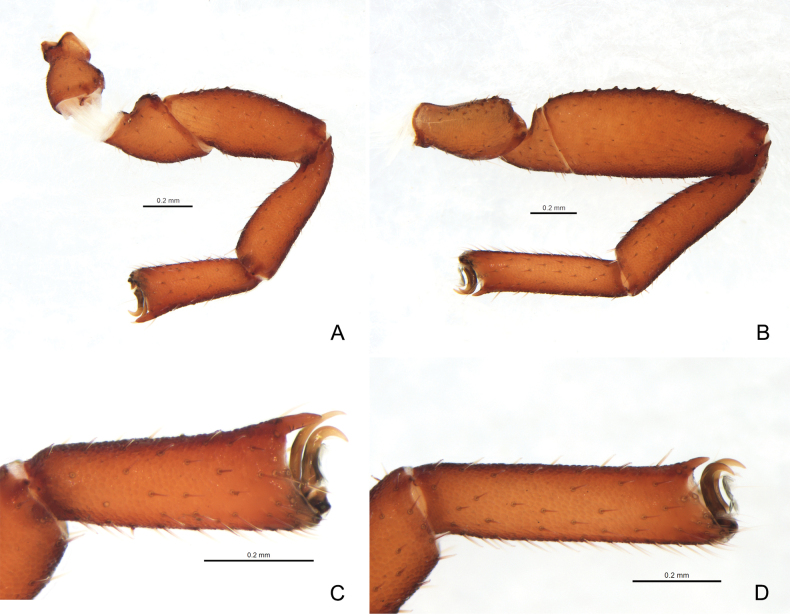
*Ellingseniusrenae* sp. nov., holotype male **A** left leg I, lateral view **B** left leg IV, lateral view **C** left tarsus I, lateral view **D** left tarsus IV, lateral view.

**Figure 5.**
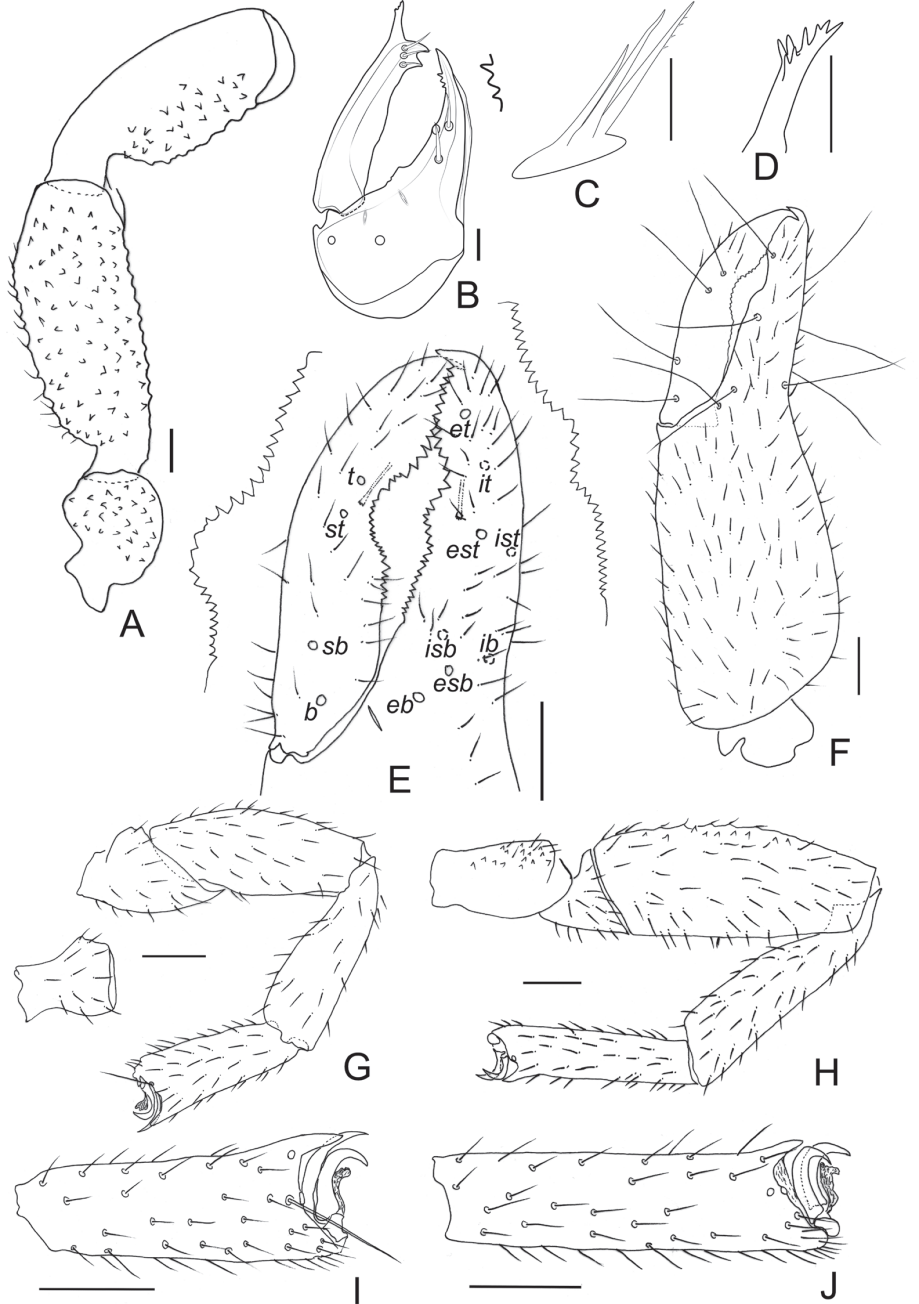
*Ellingseniusrenae* sp. nov., holotype male **A** left pedipalp, minus chela, dorsal view **B** left chelicera, dorsal view **C** rallum **D** galea **E** left chelal fingers, lateral view, showing trichobothrial pattern and teeth **F** left chelal, lateral view **G** left leg I, lateral view **H** left leg IV, lateral view **I** tarsus I, lateral view **J** tarsus IV, lateral view. Scale bars: 0.05 mm (**B–D**); 0.2 mm (**A, E–J**).

Figs 1 , 2 , 3 , 4 , 5

##### Type material.

***Holotype***: China • ♂; Ps.-MHBU-HBUARA#GZ23122701, Huohua Town, Ziyun Miao and Buyei Autonomous County, Anshun City, Guizhou Province; 25°37'46"N, 105°59'12"E; 27 November 2023, Xiaoxiao Ren leg.; collected in bee hives of *Apisceranacerana*. ***Paratype***: • 6 ♂♂ Ps.-MHBU-HBUARA#GZ23122702–07; same data as holotype • 5 ♀♀, Ps.-MHBU-HBUARA#GZ23122708–12, same data as holotype.

##### Diagnosis.

The new species is distinguished from other members of the genus *Ellingsenius* by the following combination of characters: posterior disc of carapace and tergites I–X with wrinkled surface and lateral keels; both transverse furrows on carapace prominent; carapace, pedipalpal trochanter, femur and retrolateral surface of petella with developed tubercles; middle teeth of both pedipalpal fingers concave outwards, forming a large gap; *gs* of cheliceral movable finger tripled; coxal sac and atrium absent; tarsi with dorsal projection; tarsus IV without tactile seta.

##### Etymology.

The specific epithet is a patronym in honor of Dr Xiaoxiao Ren, who collected the specimens. It is a noun in the genitive.

##### Description.

**Adult male** (Fig. 2A)

***Color***: Carapace, pedipalps, and tergites reddish brown; remaining parts (legs, sternites, and pleural membranes) yellowish brown.

***Cephalothorax*** (Fig. 3A): carapace barely longer than wide (0.91–0.95×), with a pair of eyespots; both transverse furrows prominent, distance between posterior furrow and posterior margin slightly shorter than that between posterior furrow and anterior furrow; carapace strongly granulate and with scattered larger setiferous tubercles; anterior margin with 10, posterior margin with 7–8 setae; anterior disc with c. 140, median with 75–76 and with 28–30 (243–246 in total) dentate setae, and those of anterior and median discs set on large tubercles; posterior disc with wrinkled modification; setae of carapace and tergites short and denticulate; posterior margin of carapace and tergites I–X with sclerotic lateral keels.

***Chelicera*** (Fig. 5B): chelicera small, with two acuminate setae and two lyrifissures on hand, 1.43–1.46× as long as broad, movable finger with three short, curved subdistal seta, *b* and *sb* dentate, *gs* of movable finger tripled; fixed finger with 3–4 continuous, pointed teeth; apex of movable finger with one developed tooth; serrula exterior with 27–29 lamellae; lamina exterior present; rallum (Fig. 5C) composed of three blades, distalmost blade slightly dentate; galea (Fig. 5D) relatively long and simple, with 7–8 small, distal rami.

***Pedipalp*** (Figs 3B, C, 5A, E, F): stout; all segments evenly granulated, except for smooth chelal fingers; trochanter, femur (dorsal and ventral), ventral side of patella, and hand adorned with scattered, setiferous tubercles; all setae denticulate. Apex of pedipalpal coxa with 3–4 setae, including one long seta. Chelal fingers stout; movable finger with 26–28 teeth; fixed finger with 29–32 teeth; distal ones larger, middle teeth of both fingers concave outwards, forming a large gap; venom apparatus present in both fingers; nodus ramosus (Fig. 5E): close to *st* on movable finger and to *est* on fixed finger. Trichobothrial patterns (Fig. 5E): *eb* and *esb* basally situated; *est* and *ist* medially situated; *t* far from apex of movable finger; *st* closer to *t* than to *sb*; distance between *sb* and *b* somewhat longer than distance between *esb* and *eb*. Proportions (length to breadth): trochanter 1.41–1.49×; femur 2.55–2.80×; patella 2.47–2.51×; chela with pedicel 3.20–3.39×, without pedicel 2.90–3.10×; hand with pedicel 1.80–2.00×, without pedicel 1.52–1.69×. Proportion of movable finger 0.75–0.78× as long as hand with pedicel, and 0.89–0.92× without pedicel.

***Opisthosoma***: all tergites divided by narrow, obvious median line; each half of tergites with 1–4 lyrifissures and 9–14 short, dentate setae on posterior margin, with finely granulated and wrinkled surface; tergite XI without pseudotactile seta and wrinkled modification. Coxa I with 18–21, II 19–20, III 30–32, IV with 45–50 setae. Coxal sacs of male vestigial; atrium absent. Sternites narrowly divided, with fine granulation, each half-sternites with 1–5 lyrifissures and 7–12 setae, all setae short and denticulate. Pleural membrane with irregular longitudinal grooves. Posterior margin of anterior genital operculum with 17–19 setae; posterior genital operculum with 29–34 forwardly projecting setae.

***Legs*** (Figs 4A–D, 5G–J): legs generally typical, stout. Legs I–IV covered with coarse granulation. Setae of leg I short and denticulate. Leg I: trochanter 1.33–1.74×, femur 1.74×, patella 2.58–2.70×, tibia 2.59–3.26×, tarsus 2.58–2.84× longer than deep. Tarsi with dorsal projection; tarsus I modified terminally, claws asymmetrical: anterior claw almost rectangular-curved; posterior claw slender and acute. Leg IV with short and denticulate setae. Tarsus IV without tactile setae, claws symmetrical, arolium slightly shorter than claws; subterminal setae long and simple. Trochanter 1.52–1.96×, femur + patella 2.95–3.24×, tibia 3.83–4.23×, tarsus 3.76–4.56× longer than deep.

***Dimensions*** (length/width or, in the case of the legs, length/depth in mm; ratios in parentheses). Body length 3.21–3.57. Chelicera 0.33–0.35/0.23–0.24. Carapace 1.21–1.27/1.28–1.39. Pedipalp: trochanter 0.62–0.64/0.43–0.44; femur 1.30–1.43/0.51; patella 1.21–1.23/0.49; chela with pedicel 1.92–2.00/0.59–0.60; length of chela without pedicel 1.74–1.83; length of hand with pedicel 1.08–1.18, without pedicel 0.91–1.00; length of movable finger 0.84–0.89. Leg I: trochanter 0.32–0.47/0.24–0.27; femur 0.47/0.27; patella 0.67–0.73/0.26–0.27; tibia 0.62–0.70/0.19–0.27; tarsus 0.49–0.54/0.19. Leg IV: trochanter 0.50–0.51/0.26–0.33; femur + patella 1.12–1.23/0.38; tibia 0.88–0.93/0.22–0.23; tarsus 0.64–0.73/0.16–0.17.

**Female paratype** (Figs 2B, 3F). Color slightly lighter than males. Chelicera 1.43–1.48× as long as broad. Carapace slightly longer than wide (0.80–0.93×); chaetotaxy of carapace: anterior margin with 10, posterior margin with 7–8 denticulate setae; a total of c. 260 setae. Posterior margin of carapace and tergites I–IX with vestigial lateral keels, all tergites and sternites IV–XI narrowly divided; each half tergites with 1–3 lyrifissures and 10–15 short and dentate setae on posterior margin, with fine granulation and wrinkled skin; tergite XI without pseudotactile seta and wrinkled modification; each half–sternites with 2–3 lyrifissures and 6–11 setae, all setae short and denticulate, all galea broken.

Proportions of pedipalp: trochanter 1.27–1.28×; femur 2.77–2.93×; patella 2.64–2.67×; chela with pedicel 3.50–3.51×, without pedicel 3.20–3.25× as long as broad. Hand with pedicel 1.91–1.93×, without pedicel 1.63–1.65× as long as broad. Movable finger 0.84× as long as hand with pedicel, 0.97–0.99× without pedicel. Leg I: trochanter 1.21–1.24×; femur 1.57–1.59×; patella 2.54–2.67×; tibia 3.05×; tarsus 3.13–3.43× longer than deep. Leg IV: trochanter 1.42–1.47× longer than deep; femur + patella 3.37–3.39× longer than deep; tibia 4.05–4.09× longer than deep; tarsus 4.06–4.24× longer than deep.

Body length 3.34–3.36. Chelicera 0.33–0.34/0.23. Carapace 1.23–1.24/1.32–1.55. Pedipalp: trochanter 0.57–0.64/0.45–0.50; femur 1.30–1.32/0.45–0.47; patella 1.19–1.23/0.45–0.46; chela with pedicel 1.89–1.93/0.54–0.55; length of chela without pedicel 1.73–1.79; length of hand with pedicel 1.04–1.05; length of hand without pedicel 0.88–0.91; length of movable finger 0.87–0.88. Leg I: trochanter 0.29–0.31/0.24–0.25; femur 0.44–0.46/0.28–0.29; patella 0.71–0.72/0.27–0.28; tibia 0.61–0.64/0.20–0.21; tarsus 0.48–0.50/0.14–0.16. Leg IV: trochanter 0.53–0.54/0.36–0.38; femur + patella 1.28–1.29/0.38; tibia 0.89–0.90/0.22; tarsus 0.69–0.72/0.17.

##### Distribution.

Known only from the type locality.

##### Remarks.

*Ellingseniusrenae* sp. nov. is morphologically most similar to *E.indicus* Chamberlin, 1932, as they share the following characters: tarsi with dorsal projection, coxal sacs of male vestigial, atrium absent, and similar trichobothrial pattern. The new species can be distinguished in having tergites I–IX with lateral keels in *E.indicus*, while they are with sclerotic lateral keels in *E.renae*. Pedipalps are slender in *E.renae* (femur 2.55–2.80× vs 2.30–2.40× in *E.indicus*), the carapace has a distinct longitudinal furrow in *E.indicus* (absent in *E.renae*), and the pedipalpal fingers have a larger gap in the new species (Fig. 3B; Chamberlin 1932, 1949). Furthermore, the phylogenetic analyses indicated that our samples belong to a distinct species.

### ﻿Molecular analyses

All sequences have been deposited in GenBank, with the accession numbers of the DNA barcodes provided in Table 1. The K2P genetic distance of intraspecific and interspecific nucleotide divergences of eight sequences of *Ellingsenius* are shown in Table 2.

**Table 1.** Voucher specimen and sequences information.

Species	Voucher code	Sex	GenBank accession number	Collection localities	Source
*Ellingseniusrenae* sp. nov.	ZZG001	Female	PQ730040	China, Guizhou	This study
*E.renae* sp. nov.	ZZG002	Male	PQ730041	China, Guizhou	This study
*E.* sp.	–	–	MK722157	China, Anhui	Lin et al. (2020)
*E.* sp.	–	–	MK722156	China, Anhui	Lin et al. (2020)
*E.ugandanus*	–	–	KU755526	Kenya	Fombong et al. (2016)
*E.indicus*	–	–	KT354340	Nepal	Harvey et al. (2015)
*E.ugandanus*	–	–	KU755528	Kenya	Fombong et al. (2016)
*E.ugandanus*	–	–	KU755527	Kenya	Fombong et al. (2016)
*Chelifercancroides*	–	–	OR601911	Greece	Just et al. (2023)
*Hysterochelifertuberculatus*	–	–	OR601885	France	Just et al. (2023)

**Table 2.** Intraspecific and interspecific nucleotide divergences for eight sequences of *Ellingsenius*, using Kimura 2-parameter model.

Species	MK722157	MK722156	KU755526	KT354340	KU755528	KU755527	PQ730040	PQ730041
MK722157*E.*sp.								
MK722156*E.* sp.	0.008							
KU755526 *E.ugandanus*	0.172	0.179						
KT354340 *E.indicus*	0.148	0.148	0.152					
KU755528 *E.ugandanus*	0.172	0.179	0.000	0.152				
KU755527 *E.ugandanus*	0.172	0.179	0.000	0.152	0.000			
PQ7300410*E.renae* sp. nov.	0.005	0.003	0.158	0.124	0.158	0.158		
PQ730041*E.renae* sp. nov.	0.008	0.005	0.156	0.126	0.156	0.156	0.002	

Although the specimens of *Ellingseniusrenae* (ZZG001, ZZG002) were collected at localities > 1100 km away from the specimens reported by Lin et al. (2020) (MK722156, MK722157), they have a relatively low genetic distance (0.3–0.8%), which was much lower than the interspecific genetic distance in other species (12.4–17.9%) (Table 2) in the dataset. Consequently, we consider *E.renae* and Lin et al.’s (2020) undescribed species to be conspecific.

The intraspecific genetic distance ranged from 0–0.8%, and the interspecific genetic distance ranged from 12.4–17.9%. All maximum intraspecific distances were much lower than minimum interspecific distances for all species in this study and the optimal identification threshold of 4.7% for Chthoniidae and 3.6% for Neobisiidae (Hlebec et al. 2023). The results of Kimura 2-parameter genetic distances confirm the associated matching of male and female in our dataset.

**Figure 6.**
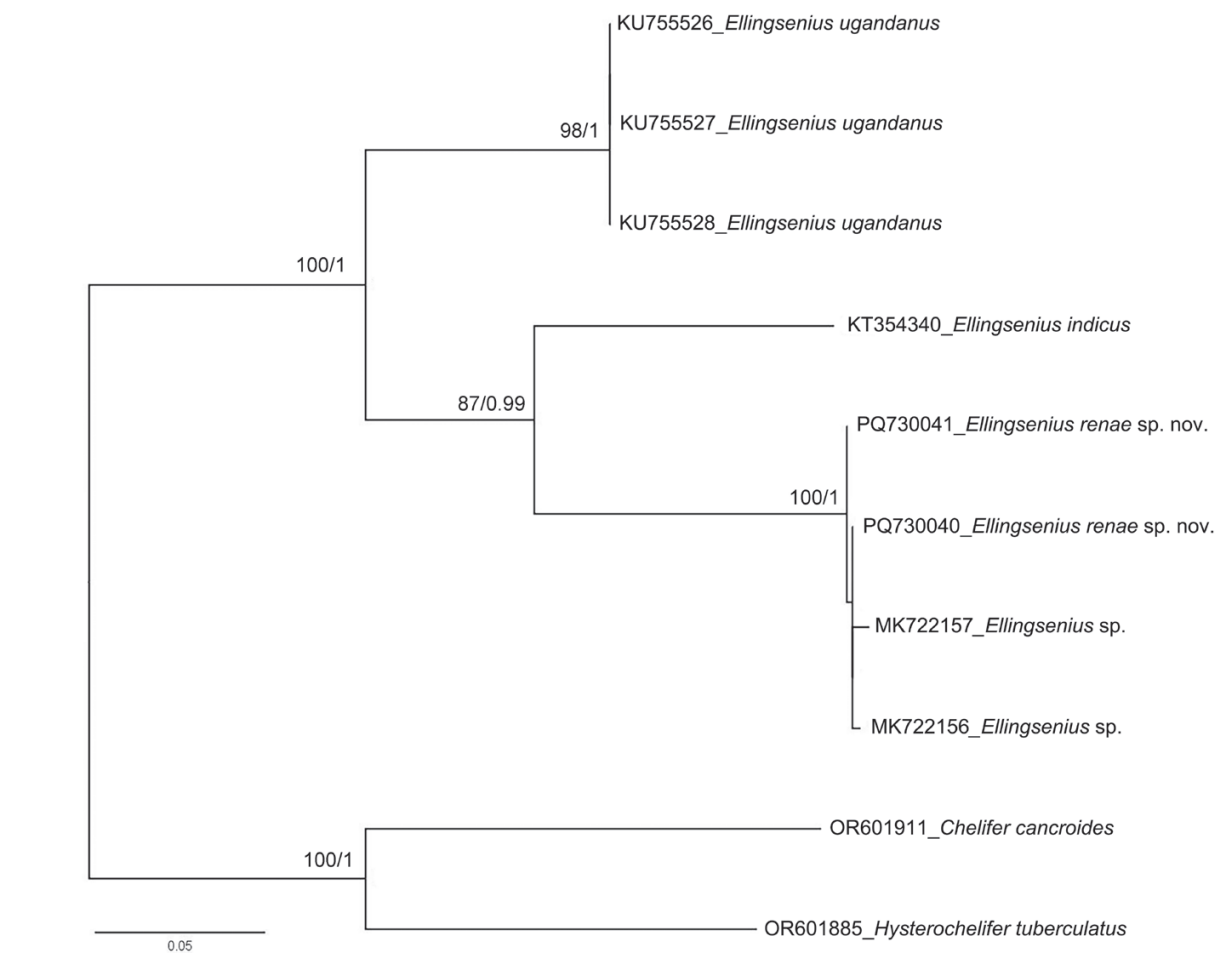
ML phylogram based on *COI* sequence data. The topology is equivalent in both the ML and BI analyses. Support for each node is represented by ultrafast bootstrap values (uBV, %) and posterior probability (pp).

The ML and BI analyses result in the same relationships for the *Ellingsenius* clade (Fig. 6). The topology of the ML tree (Fig. 6), equivalent to that of the BI phylogram, showed three clades representing three *Ellingsenius* species. The monophyly of *E.indicus* and *E.renae* were strongly supported (uBV = 87%, pp = 0.99; uBV = 100%, pp = 1) and *E.ugandanus* was the sister group to *E.indicus* and *E.renae* (uBV = 98%, pp = 1). The present phylogenetic analyses result also support *E.renae* and *E.* sp. (MK722156, MK722157) as being conspecific.

### ﻿Key to species of *Ellingsenius*

**Table:** 

1	Chelal hand with many well-developed tubercles	**2**
–	Chelal hand with few vestigial tubercles or without tubercles	**3**
2	Tergites smooth, carapace with vestigial transverse furrows	***E.perpustulatus***
–	Tergites strongly granulate and sculptured, carapace with prominent transverse furrows	***E.hendrickxi***
3	Pedipalps stout, femur < 2.5× and patella < 2.0× longer than broad	**4**
–	Pedipalp slender, femur > 2.5× and patella > 2.0× longer than broad	**5**
4	Chelal fingers longer than hand	***E.globosus***
–	Chelal fingers clearly shorter than hand	***E.indicus***
5	Tarsus of legs with well-developed dorsal projections	***E.renae***
–	Tarsus of legs with vestigial or without dorsal projections	**6**
6	All surfaces of pedipalpal femur and patella with tubercles	***E.sculpturatus***
–	Only prolateral surface of pedipalpal femur and patella with tubercles	**7**
7	Pedipalpal femur and patella with few well- developed tubercles	***E.ugandanus***
–	Pedipalpal femur and patella with a larger number of vestigial tubercles	***E.fulleri***

## ﻿Discussion

**Figure 7.**
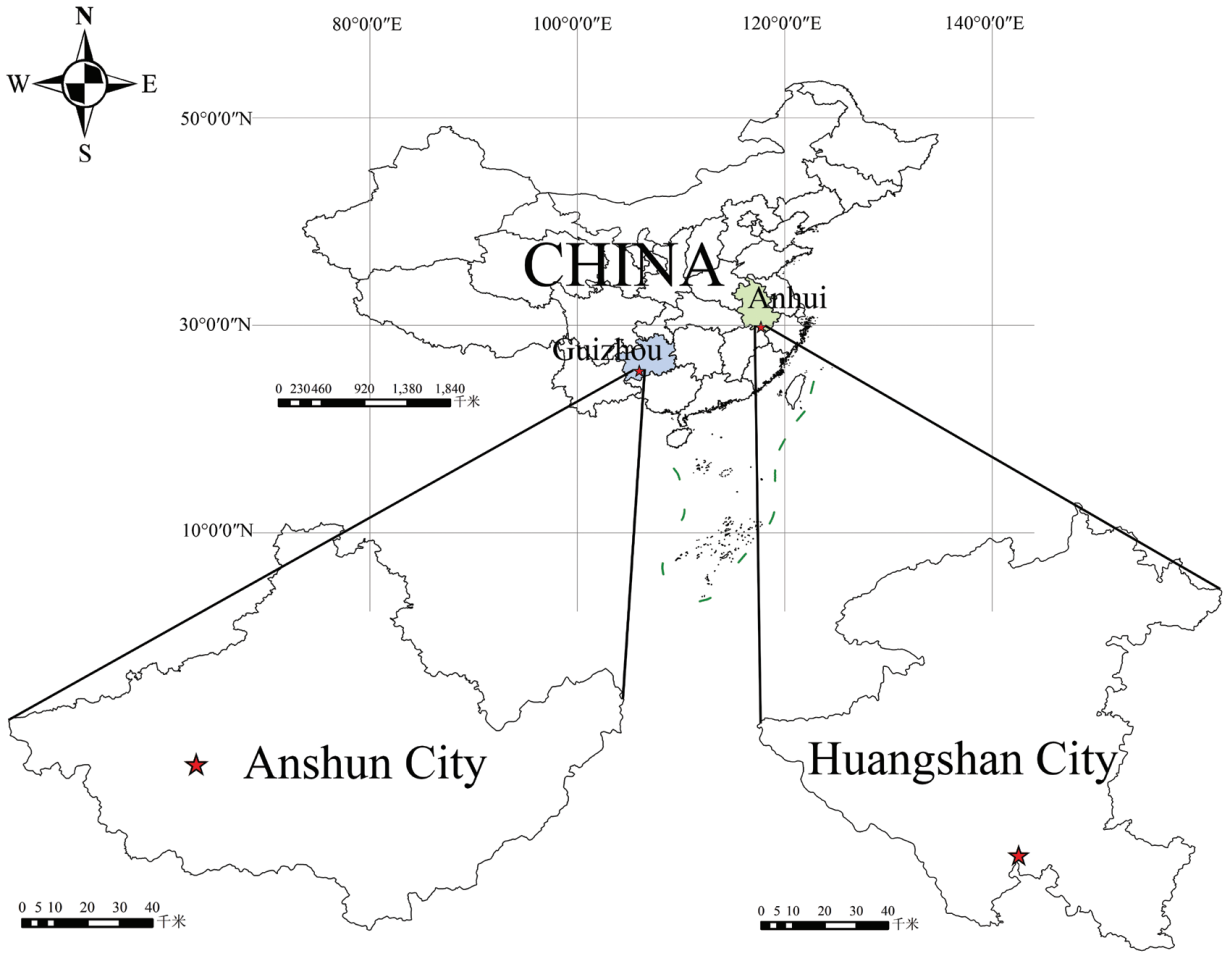
Known distribution areas of *Ellingseniusrenae* sp. nov. (Lin et al. 2020).

Fifteen pseudoscorpion species, belonging to six genera in three families, have been reported from colonies of three stingless bee species and two honeybee species, and all *Ellingsenius* species occur as commensals in beehives (Gonzalez et al. 2008). *Ellingseniusrenae* sp. nov. was found in the beehives of *Apisceranacerana* from southern China (Fig. 7), similar to *E.indicus*, which is also associated with *A.c.cerana* (Gonzalez et al. 2008).

Pseudoscorpions are considered beneficial to bees because they eat *Varroa* mites and other pests of bees (Donovan and Paul 2005, 2006; van Toor et al. 2015). However, *E.hendrickxi* Vachon, 1954 preys on the host bees (Vachon 1954), negating their usefulness to apiarists. Based on the observations of apiarists from Guizhou, China, *E.renae* in beehives are usually harmless to the bees; on the contrary, they prey on bee mites that parasitize the beehive. Once *E.renae* individuals appear in a beehive, they are usually found in relatively low numbers, and bees do not attack them. Subsequently, the number of *E.renae* will gradually form a certain population size, and if the number of *E.renae*is too high in a hive, it may affect the activity of the bees. The bees may attempt to drive *E.renae* away, and in this case, *E.renae* will disperse to other hives through phoresy on the bees.

Phoretic behavior is commonly found in pseudoscorpions (Muchmore 1971; Poinar et al. 1998), especially in the Cheliferidae. The characteristic gap in the middle of the chelal fingers in both males and females of *E.renae***sp. nov.** (Figs 3B, 5E) may be evidence of phoretic behavior on bees, as pseudoscorpions in the beehive will grasp the legs of bees for phoresy, with the gap allowing a firmer grip.

The ecology of pseudoscorpions in beehives suggests that they have potential as biological control agents of bee pests, especially in controlling bee mites. If pseudoscorpions can effectively control these pests, beekeepers would not need to use chemical agents, which would avoid chemical residues in honey or wax, benefit the environment, and may also avoid the mites’ resistance to evolution.

## Supplementary Material

XML Treatment for Ellingsenius

XML Treatment for Ellingsenius renae

## ﻿Additional information

### Author ORCIDs

Zhizhong Gao https://orcid.org/0000-0002-6666-8746

Jianzhou Sun https://orcid.org/0009-0001-4940-7130

Feng Zhang https://orcid.org/0000-0002-3347-1031

### Data availability

All genetic data have been deposited in GenBank. The final alignment are provided online as supplementary files.

## Supplementary materials

Supplementary material 1 Sequence alignment Data type fas https://binary.pensoft.net/file/1305947This dataset is made available under the Open Database License (http://opendatacommons.org/licenses/odbl/1.0/). The Open Database License (ODbL) is a license agreement intended to allow users to freely share, modify, and use this Dataset while maintaining this same freedom for others, provided that the original source and author(s) are credited.Zhizhong Gao, Jianzhou Sun, Feng Zhang

Supplementary material 2 Sequence alignment https://doi.org/10.3897/zookeys.1234.144259.suppl2 Data type fas https://binary.pensoft.net/file/1305948This dataset is made available under the Open Database License (http://opendatacommons.org/licenses/odbl/1.0/). The Open Database License (ODbL) is a license agreement intended to allow users to freely share, modify, and use this Dataset while maintaining this same freedom for others, provided that the original source and author(s) are credited.Zhizhong Gao, Jianzhou Sun, Feng Zhang
